# The Influence of Socioeconomic and Demographic Variables on Willingness to Donate Cadaveric Human Organs in Malaysia

**DOI:** 10.1097/MD.0000000000000126

**Published:** 2014-11-14

**Authors:** Rajah Rasiah, Rishya Manikam, Sankara K. Chandarsekaran, Govindamal Thangiah, Saravanan Puspharajan, Dasan Swaminathan

**Affiliations:** Department of Development Studies (RR, GT); Department of Emergency and Trauma (RM); Department of Orthopaedic Surgery (SKC); Department of Restorative Dentistry (SP); and Department of Dentistry (DS), University of Malaya, Lembah Pantai, Kuala Lumpur, Wilayah Persekutuan Kuala Lumpur, Malaysia.

## Abstract

The growing shortage in human organs has raised serious concerns. To address this problem, we examine in this article the association between demographic and socioeconomic factors, and respondents’ willingness to donate cadaveric organs using a large survey of Malaysian adults aged 18 years and above.

A convenience sampling method was used to extract information from a total of 10,350 participants from Metropolitan Kuala Lumpur over the period of April 2, 2013 to February 29, 2014. In addition to analyzing the data using incidence of willingness to donate by demographic and socioeconomic factors, we carried out logistic regression analysis to estimate the odds ratio of respondents’ willingness to become cadaveric organ donors controlling for age.

About less than a third of the participants pledged to donate their organs upon death with women (35.6%) showing a higher incidence compared with men (33.2%). The Chinese (35.7%) and Malays (35.0%) pledged to contribute more than the Indians (31.6%) and the logistic regressions show that Malays (adjusted odds ration [OR] = 1.18) and Chinese (adjusted OR = 1.21) are more likely to donate than Indians (reference group). The results by religion were significant among Muslims and Hindus but not Buddhists. The likelihood of Muslims donating was the lowest (adjusted OR = 0.26). Income was also highly significant but the relationship with willingness to donate was negative. Against tertiary education, all other occupations were significant. However, the respondents with primary education enjoyed the highest adjusted OR (5.46) whereas that of secondary (0.48) and higher secondary (0.83) education was low. Among occupations (against supervisory, clerical, and direct workers), it was significant only among the unemployed and managers with adjusted OR of 1.50 and 1.58, respectively.

Sex, education, ethnicity, religion, and income are important demographic and socioeconomic influences on the likelihood of Malaysians willing to become cadaveric organ donors. The Malaysian evidence suggests that awareness programs should be targeted at men, Muslims, Hindus, Malays, and the rich more than the others.

## INTRODUCTION

Efforts by governments to increase organ donation in several countries have borne little results.^1^ Cadaveric organ donations (that refer to harvest of organs from deceased donors and their transfer to recipients) only came to 0.5 persons/million people in Malaysia in 2012.^2^ Also, the waiting list for kidney transplants in Malaysia, for example, rose from 5542 in 1999 to 15,489 in 2012.^3^

Understandably, the deficit in the supply of human organs for transplantation prompted the Malaysian government to explore various initiatives, including the launching of a policy on unrelated living organ donation in 2007 to address the shortage (Article 6.2.3),^4^ which was strengthened in 2011 by making the procedures more specific.^5^ However, this article focuses on only cadaveric organ donations in light of the concerns over the dangers faced by living donors, which have been raised by the World Health Organization (WHO), including international organ trafficking and medical tourism. For these reasons, the WHO has discouraged the pursuit of living donations and instead called for the maximization of the therapeutic potential of cadaveric donations.^6,7^

Although there are important initiatives calling for greater awareness creation to encourage people to become cadaveric donors,^6^ existing works targeting the demographic socioeconomic background of potential donors lack consensus. Some studies show that the Chinese, both in the mainland and abroad–influenced by Confucian, Buddhist, Daoist, and other spiritual values–are generally unwilling to donate organs.^8–10^ Studies comparing donor conduct of blacks and whites on willingness to donate present contradictory results: 1 study found statistically significant difference by age but not by ethnicity,^11^ whereas another found ethnicity, religion, and sex to be important.^1^ Although some studies reported that Asians are less reluctant to be donors than whites, another study reported that the Chinese (57.0%) have pledged most to be organ donors followed by Indians (22.0%), Malays (11.4%), and other ethnic minorities (2.7%). This study also reported the breakdown by ethnicity among actual donors: Chinese 57%, Indians 33%, and Malays 7%. Using a sample of 105 respondents, a study on Malaysia showed that Chinese Buddhists are encouraged by their religion to donate whereas Malay Muslims and Indian Hindus are prohibited by their religions to donate organs.^12^

There is also a lack of consensus on the influence of the socioeconomic variables of education and income on the willingness to donate cadaveric organs. One study on Malaysia and another on Europe found education to be positively correlated with willingness to donate.^13,14^ However, another study did not find any statistical link between education and the willingness to donate.^15^ Although 1 study showed an inverse relationship between income and willingness to donate in the United Kingdom,^16^ another study reported the opposite in Canada.^17^

The focus on only cadaveric rather than living donors is particularly important because the Oviedo Declaration, the Strasbourg Protocol, and the Istanbul Declaration prohibit financial compensation as they could lead to the exploitation of the poor through international trafficking in human organs.^6^

The multiethnic and multireligious population of Malaysia provides an excellent example to reexamine the hypothesis that sex, ethnicity, and religion on the one hand and education and income on the other hand may have a strong influence over the willingness of people to donate cadaveric organs. The findings may generate strong policy implications to ameliorate the problem of growing deficit between demand and delivery of human organs.

## MATERIALS AND METHODS

Following a request by the head of the organ transplant unit at the Ministry of Health in Malaysia to expand the register of Malaysians willing to donate their organs upon death, we applied for a grant to support this cause with the intention of expanding it to cover the whole country after learning from this experience. One of the conditions of the grant was for us to publish articles in high impact journals, and hence, this article. The study uses a survey from the Federal Territory of Kuala Lumpur. A pilot study using a questionnaire and face-to-face interviews with 100 persons drawn from the telephone registry of people in Kuala Lumpur was undertaken to help us understand the population and test the robustness of the questionnaire with people. The organs we referred to are heart, liver, and kidney. We found that selecting a randomly stratified sample will be difficult because 29 persons could not be reached at their home phones despite attempts to call them once in the morning, once in the afternoon, and once between 7 and 9 pm. Thirty three persons refused to participate in the survey. Hence, we chose to use the convenience sampling method in which we selected 5 hypermarkets, 5 government hospitals, 2 universities (1 public and 1 private), and 2 locations with large concentrations of shopping and commercial operations. The study was approved by the University of Malaya ethics committee. The survey was led by 2 researchers who used a team of 6 enumerators each to approach all persons and to explain to them the purpose of the survey and why their participation will be helpful for the country. In doing so, we followed the procedure that other major studies have used to ensure a high response rate when the study involves seeking sensitive information.^18^ Information of all Malaysians aged 18 and above, who voluntarily agreed to participate in the survey, were recorded. The breakdown of the sample is shown in Table 1.

**TABLE 1:**
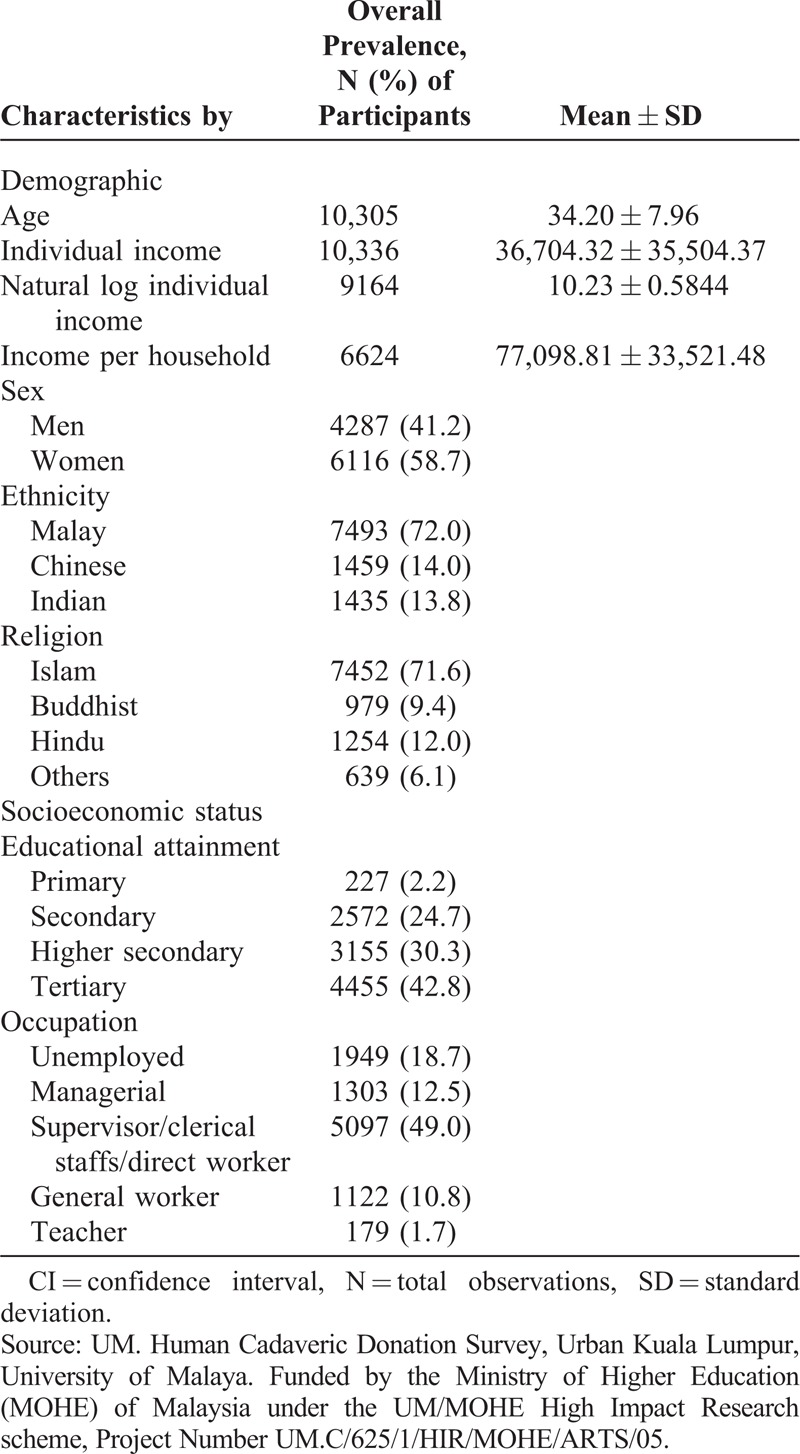
Socioeconomic and Demographic Characteristics in the Sample

Because some individuals refused to answer certain questions (eg, incomes and education levels), the response rates varied with each of the questions, but they all exceeded 70%. The use of medical professors and students as coordinators and enumerators, who carried their designation cards with them, helped to raise the response rate. The survey lasted from April 2, 2013 till February 29, 2014, netting 10,350 respondents in total. The breakdown of the sample by sex was 41.3% men and 58.7% women, by ethnicity was 72.1% Malays, 14.0% Chinese, and 13.8% Indians, and by religion was 72.4% Muslims, 9.4% Buddhists, 12.2% Hindus, and 6.1% other religions. We dropped 24 observations from East Malaysians, Portuguese, and Thais as the numbers were too small. We combined Indians with others because the number of Indian respondents was small. The others category included mainly Christians and also free thinkers.

We used both descriptive statistics and multiple logistic regressions for the analytic methodology with the latter controlling for other effects. The large sample helped generate some robust results for interpretation.

## RESULTS

The percentages of respondents who agreed to donate their organs upon death by sex, ethnicity, religion, education, and occupation are shown in Table 2. Overall, less than a third of the respondents registered to donate their organs upon death. The share was slightly higher among women (35.6%) compared with men (33.2%), and the results are significant at the 1% level of significance. Among the 3 ethnic groups, shares among the Chinese (35.7%) and Malays (35.0%) were higher than Indians (31.6%) but only the last was statistically significant. The simple statistical analysis showed highly significant results by religion. Others (58.1%) and Buddhists (53.7%) recorded the highest shares among those willing to donate organs upon death. The lowest shares were recorded among Muslims (27.7%) and Hindus (45.8%).

**TABLE 2:**
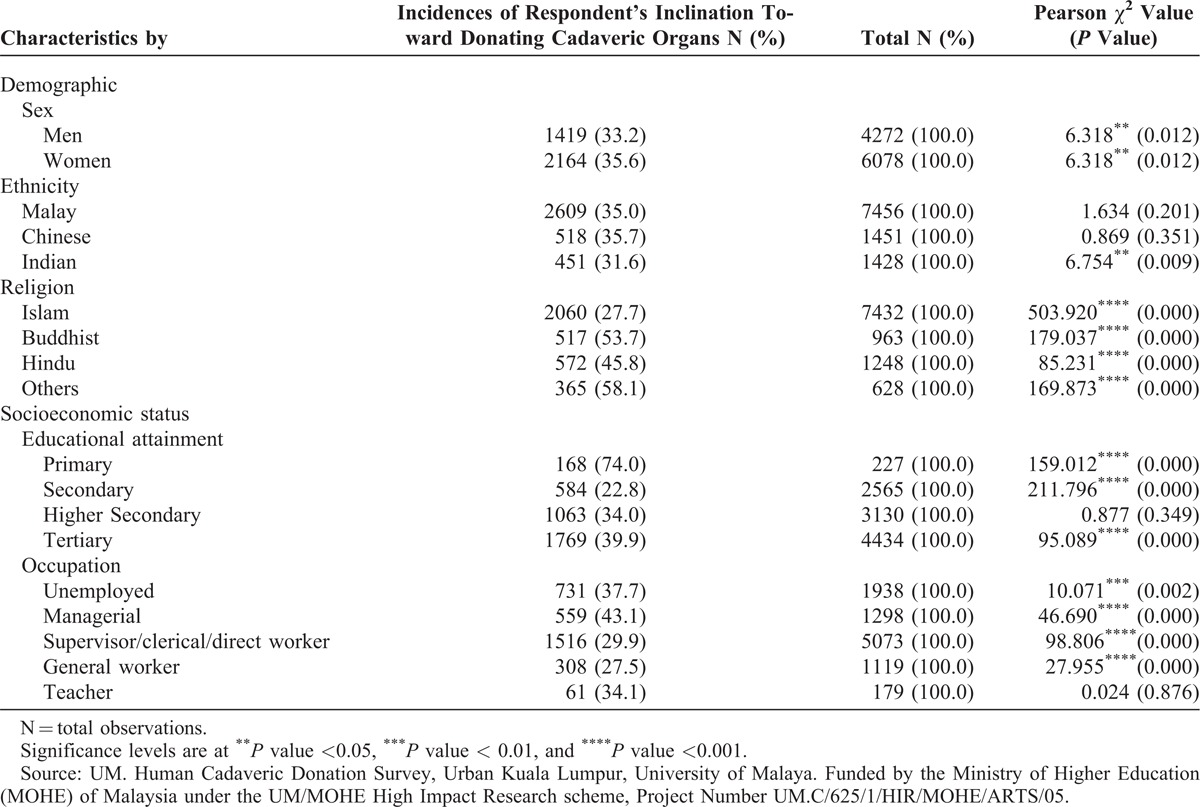
Incidences of Respondents Agreeing to Donate Cadaveric Human Organs

The analysis by education levels showed that the highest incidence of those wanting to donate was among the primary education holders (74.0%) followed by tertiary (39.9%), higher secondary (34.0%), and secondary level education holders (22.8%). Except for primary education, these results show a rising trend in incidence among education levels. These results are not surprising because those with primary education primarily constitute the low income earners, who are more willing to donate than the high income earners, among the respondents.

## DISCUSSION

The multiethnic and multireligious background of the population produced interesting results, which will be useful to draw implications for many regions. Table 3 presents the results of the multiple logistic regression results against the demographic and socioeconomic variables using the dependent dummy (1,0) variable of willingness to donate cadaveric organs. Education levels were used in this equation to remove the significance of the constant else the statistical results were biased by endogeneity problems. Age was not significant, which does not support some past findings on black and white samples in the United States.^11^ Sex was highly significant (1%) with the unadjusted and adjusted odds ratio (OR) of men at 0.90 and 0.84, respectively. Consistent with the simple statistical assessment (Table 2), the logistic regression results reinforced the findings by sex (Table 3). Clearly, women are more likely to donate organs than men even when controlled for other influences.

**TABLE 3:**
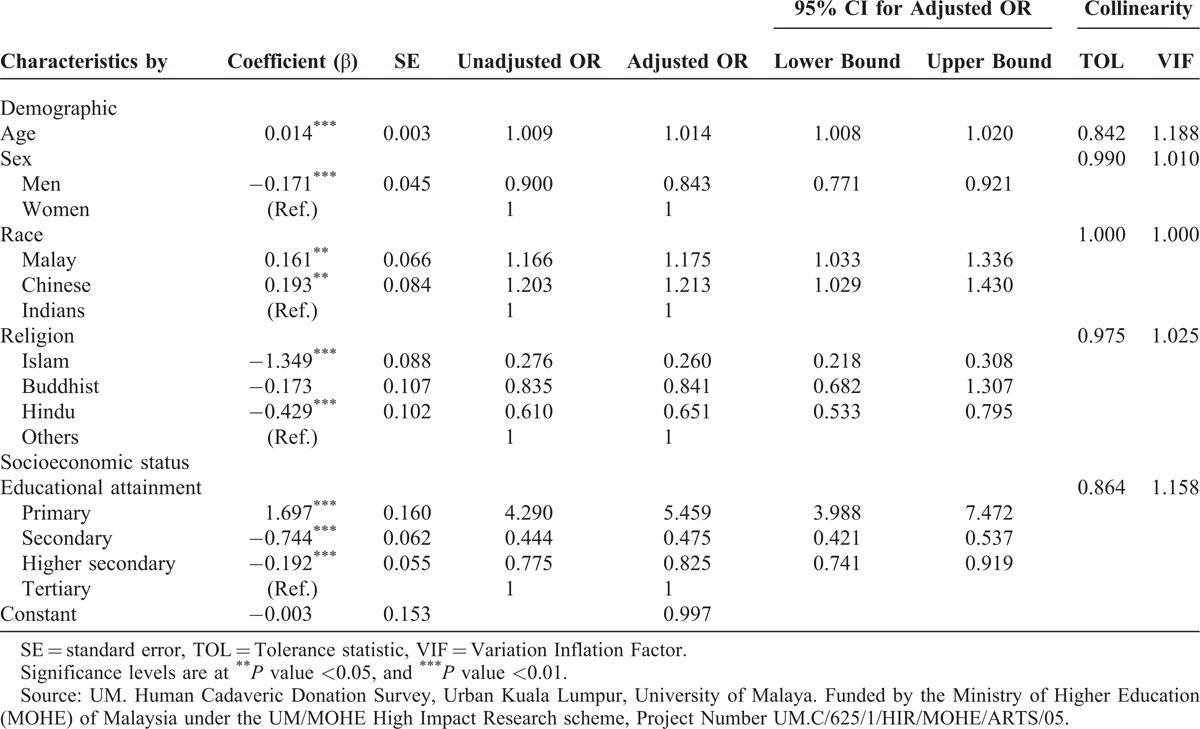
Fitted Demographic and Socioeconomic Predictors by Means of Multiple Logistic Regression Analysis

The breakdown by ethnicity was also significant with Malays and Chinese showing slightly higher ORs than Indians, which does not support a past finding on Malaysia.^12^ The religious background of the respondents was only significant with Muslims and Hindus, which is consistent with past results on Malaysia and Singapore.^12^ However, against others the OR and adjusted OR of Muslims was the lowest at 0.28 and 0.26, respectively. Hindus were next at 0.84 and 0.84, respectively. The results of Chinese Buddhists were consistent but not of Indian Hindus with a past finding on Malaysia.^12^ The OR of Buddhists was not significant. The results were the same even after controlling for age, sex, and education. All variables passed the collinearity test whereas the nonsignificant constant shows that results do not suffer from endogeneity problems.

Table 4 presents the results of multiple logistic regression results against demographic and socioeconomic variables using the dependent variable of a dummy (1,0) on the question of respondents agreeing or disagreeing with cadaveric organ donation upon death, but this time with a focus on occupational classification.

**TABLE 4:**
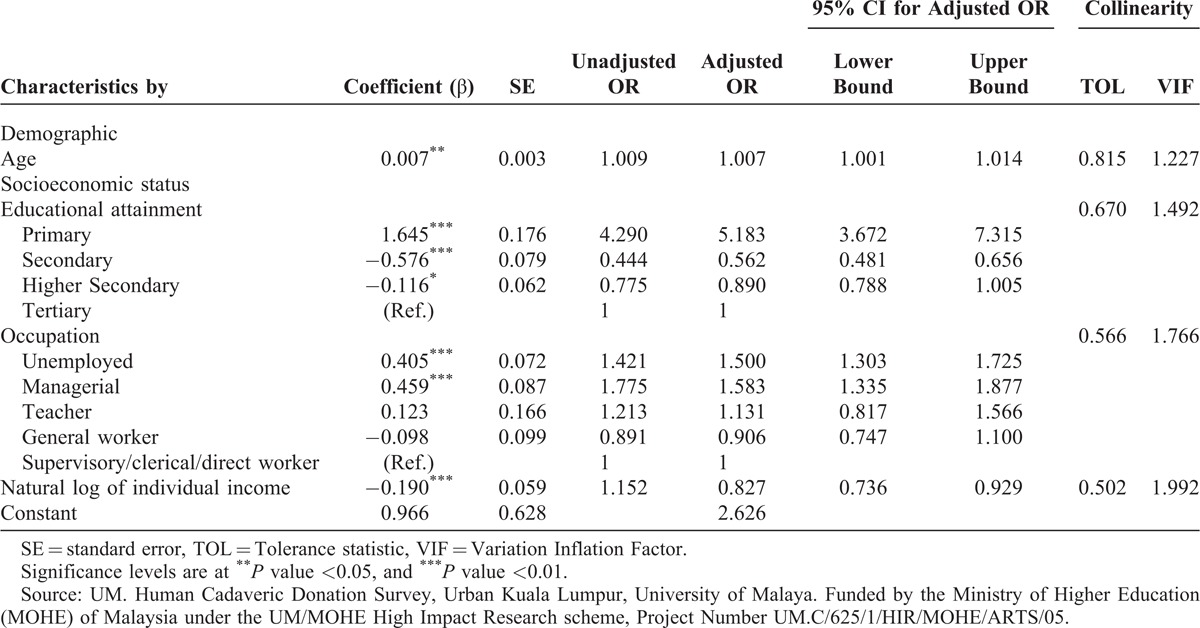
Fitted Demographic and Socioeconomic Predictors by Means of Multiple Logistic Regression Analysis

Education was highly significant and the relationship with a willingness to donate positive, which supports past findings.^13,14^ Against tertiary education as the reference variable, primary education enjoyed an OR and adjusted OR of 4.3 and 5.5, respectively. Secondary education had an OR and adjusted OR of 0.44 and 0.48, respectively. Higher secondary education had an OR and adjusted OR of 0.78 and 0.83, respectively.

Income was also highly significant with the relationship with willingness to donate negative, which is consistent with the findings in the United Kingdom^16^ but not that of Canada.^17^ The adjusted OR was 0.83, which shows that low income earners are more likely to donate than higher income earners in Malaysia.

Among the occupational categories, the results of the unemployed and managers were significant against the reference variable. The rest of the occupation levels were not significant. Income was highly significant but the coefficient was negative suggesting that the poor in Malaysia are more inclined to donate their organs upon death than the rich. The OR and adjusted OR of income taken in natural logarithm was 1.15 and 0.83, respectively. The results are robust as the constant is insignificant suggesting that the regression model has no endogeneity problems.

## CONCLUSION

Overall sex, education, ethnicity, religion, and income were the most significant demographic and socioeconomic influences on the likelihood of Malaysians to donate their organs upon death. The large sample used calls for a serious assessment of the findings. The Malaysian evidence shows that it is important that awareness programs are targeted at men, Muslims, Hindus, and Malays, the rich more than the others.

## LIMITATIONS

Although the results are robust, they should be treated with some caution as the study relied on a cross-sectional rather than a panel data set, which is important to establish causality. Also, we did not attempt mediating influences on the relationships to check if the high incidence of those with primary education wanting to donate was influenced by the presence of financial incentives. Future studies should focus on following a sample over time and attempting mediation analysis.

## ACKNOWLEDGMENT

We authors would like to thank the ethics committee of the University of Malaya for endorsing this study.
